# Conjugated linoleic acid improves oocyte cryosurvival through modulation of the cryoprotectants influx rate

**DOI:** 10.1186/s12958-015-0059-3

**Published:** 2015-06-12

**Authors:** Joana E. Matos, Carla C. Marques, Teresa F. Moura, Maria C. Baptista, Antonio E. M. Horta, Graça Soveral, Rosa M. L. N. Pereira

**Affiliations:** Research Institute for Medicines (iMed.ULisboa), Faculty of Pharmacy, Universidade de Lisboa, Av. Prof. Gama Pinto, Universidade de Lisboa, 1649-003 Lisbon, Portugal; INIAV, Unidade de Biotecnologias e Recursos Genéticos, Quinta da Fonte Boa, 2005-048 Vale de Santarém, Portugal; Departamento Bioquímica e Biologia Humana, Faculdade de Farmácia, Universidade de Lisboa, Av. Prof. Gama Pinto, 1649-003 Lisbon, Portugal; Faculdade de Ciências e Tecnologia, Universidade Nova de Lisboa, Monte de Caparica, 2829-516 Caparica, Portugal; Escola Universitária Vasco da Gama, Campus Universitário de Lordemão, 3020-210 Coimbra, Portugal; CIISA, Faculdade de Medicina Veterinária da Universidade de Lisboa, Avenida da Universidade Técnica, 1300-477 Lisbon, Portugal

**Keywords:** CLA conjugated linoleic acid, Oocytes, Membrane permeability, Cryopreservation

## Abstract

**Background:**

In cryopreservation, oocytes are subjected to extreme hyperosmotic conditions, inducing large volume changes that, along with an abrupt temperature drop, interfere with their developmental competence. Our objectives in this work were to find conditions enabling an increase in oocyte cryosurvival and subsequent development.

**Methods:**

Abattoir-derived bovine oocytes were cultured without (Control group) or with trans-10,cis-12 conjugated linoleic acid isomer (CLA group). Comparative observations were made for *1)* the oocyte developmental competence after exposure to cryoprotectants followed or not by vitrification/warming, *2)* the oocyte membrane permeability to water (using the non-permeant cryoprotectant sucrose) and 3) the oocyte membrane permeability to two cryoprotectants (ethylene glycol, EG, and dimethyl sulfoxide, DMSO). Mature oocytes cultured with or without CLA and vitrified/warmed or only exposed to cryoprotectants without vitrification were subjected to in vitro fertilization; embryo culture proceeded until the blastocyst stage. The oocyte membrane permeabilities to water and cryoprotectants were estimated using mature oocytes subjected to hyperosmotic challenges. For water permeability, 200 mM sucrose was used, whereas for the cryoprotectant permeability, a 10 % solution of both EG and DMSO was used. The data were analyzed using the MIXED procedure and Student’s *T*-test.

**Results:**

CLA supplementation improves the developmental competence of vitrified/warmed and cryoprotectants exposed oocytes (*p* < 0.01) and reduces their membrane permeability to water (37 %, *p* < 0.001) and to cryoprotectants (42 %, *p* < 0.001).

**Conclusions:**

: By slowing the fluxes of water and of permeant cryoprotectants, CLA contributed to improved oocyte cryosurvival and post-thawed viability. This isomer supplementation to the maturation media should be considered when designing new protocols for oocyte cryopreservation.

## Background

Oocyte cryopreservation is increasingly in demand due to the need for preserving gametes of both humans and animals. Oocyte cryopreservation is imperative for posterior viable reproduction in women in danger of losing ovarian function, such as women suffering from cancer who need to undergo radiotherapy or chemotherapy. The value of oocyte cryopreservation is not restricted to humans; it extends to highly valuable females of any species, either for conservation or for commercial purposes. However, oocytes are sensitive to cryopreservation, and although progress has been made in the past few years, the perfect protocol is far from established [1, 2].

Cryopreservation requires several steps in which cells are subjected to extreme hyperosmotic conditions. The cumulative osmotic stress upon the cell due to water and cryoprotectant fluxes triggers abrupt cell volume changes that may compromise cell viability and cryopreservation success [3, 4]. In contrast to spermatozoa, the cryopreservation of female gametes shows a lower success rate. This problem may be partially due to the large oocyte size and shape, with a low surface to volume ratio. In addition, the permeability features of the plasma membrane directly affect the rates of water and cryoprotectant flux and, consequently, the cryopreservation process. Thus, membrane permeability plays an important cryobiological role in cell survival after vitrification [1, 3–5].

Emerging studies in the field of lipid metabolism revealed that trans-10, cis-12 conjugated linoleic acid (CLA), a dietary fatty acid frequently used as a body fat reducing agent, reduces the permeability and fluidity of adipose plasma membranes [6]. Conjugated linoleic acid consists of a collection of positional and geometrical isomers of octadecadienoic acid, with conjugated double bonds ranging from 6 or 8 to 12 or 14. Milk and other dairy products are good sources of conjugated linoleic acid isomers. Rumen bacteria have the unique ability to convert linoleic acid into CLA through an enzymatic isomerase reaction [7]. Recent reports have shown that the reproductive performance of dairy cows may be improved by feeding CLA supplements during early lactation [8]. The biological mechanisms underlying the beneficial effects of CLA on reproductive performance are not yet fully understood. We have previously stated that the addition of CLA isomer to embryo culture medium more than doubles embryo cryoresistance [9, 10]. Additionally, the presence of CLA during bovine oocyte maturation improves oocyte competence to develop into higher quality embryos, interfering with lipid metabolism [11]. However, the mechanisms resulting in these benefits remain to be elucidated.

Most often, the design of new protocols for oocyte cryopreservation is performed in an empirical way based on the observation of outcomes in terms of oocyte survival, fertilization and embryo development. However, this simple morphological analysis provides very little information on the mechanisms by which cryoprotectants and cooling rates affect the normal physiology and function of the oocyte. The objective of this work is to contribute to a better understanding of such mechanisms to provide valuable insight into the action of cryopreservation protocols. Thus, we investigated the effect of CLA on 1) oocyte developmental competence after exposure to cryoprotectants following or not vitrification/warming; 2) oocyte membrane permeability to water (using the non-permeant cryoprotectant sucrose) and to two permeant cryoprotectants (EG and DMSO).

## Methods

All chemicals used were purchased from Sigma Aldrich Chemical Co. (St. Louis, USA) unless specified otherwise.

### Oocyte collection and in vitro maturation

Young heifer oocytes aspirated from slaughterhouse ovaries with at least three layers of compact cumulus cells and an evenly granulated cytoplasm were selected for maturation [11]. Control oocytes were matured in tissue culture medium (TCM) 199 with 10 % serum, 10 μg mL^−1^ follicle stimulating hormone (FSH), 100 μM glutathione and antibiotics in an incubator at 39 °C with humidified air and 5 % CO2 during 22 h [11]. CLA oocytes were matured in the same medium supplemented with 100 μM CLA.

### Oocyte cryopreservation

Oocytes were vitrified using an open pulled straw (OPS) method previously described by Vajta et al. [12]. Before vitrification, mature oocytes with at least one layer of cumulus cells (control vitrified *n* = 261, CLA vitrified *n* = 245) were immersed for 30 s in holding medium [HM: TCM199 plus 20 % fetal calf serum (FCS)], supplemented with 10 % EG and 10 % DMSO. Oocytes were then transferred to vitrification solution (HM plus 20 % EG, 20 % DMSO and 0.5 M sucrose) for 25 s. Afterwards, COC were loaded into a modified straw (OPS) by capillarity. Straws containing the oocytes were immediately plunged into liquid nitrogen (LN2). All media and all manipulations were performed at an ambient temperature of 25 °C.

For warming, the oocytes were expelled and equilibrated for 5 min in a warming solution (0.3 M sucrose in HM) at 37 °C. Oocytes were then washed and maintained in HM. Only intact oocytes showing no signs of degeneration and with at least one layer of cumulus cells were considered viable by an expertise technician and were selected for in vitro fertilization (IVF).

In a second experiment, mature oocytes supplemented or not with CLA were exposed to cryoprotectants according to the same process as above but without loading of the oocytes in OPS and immersion in LN2.

### Oocyte fertilization and embryo culture

Oocyte fertilization was performed with frozen–thawed semen following swim-up procedures, as in [9]. The in vitro fertilization medium consisted of modified Tyrode’s medium supplemented with 5.4 USP mL^−1^ heparin, 10 mM penicillamine, 20 mM hypotaurine and 0.25 mM epinephrine. The sperm concentration was adjusted to 10^6^ spermatozoa mL^−1^. Sperm and oocytes were co-incubated for 22 h (IVF = day 0). Presumptive zygotes were placed into synthetic oviductal fluid (SOF) supplemented with BME and MEM amino acids and bovine serum albumin (BSA) and were cultured at 39 °C in a humidified atmosphere with 5 % O2, 5 % CO2 and 90 % N2. After assessing cleavage at 48 h post insemination, embryo development proceeded in SOF plus 10 % FCS. Cleavage and day 7/8 embryo rates were calculated considering only viable oocytes. Morphological scoring of day 7/8 embryos was performed based on morphological criteria and the developmental stage. Briefly, following the guidelines of the International Embryo Transfer Society [13], embryos were classified into 3 categories: 1) Grade 1: Good, no blemishes or only trivial imperfections; 2) Grade 2: Fair, with some extruded or degenerated cells and non-uniform, darker appearance; and 3) Grade 3: Bad, poor quality, lacking cohesion or with many extruded or degenerated cells.

### Oocyte membrane permeability to water and to cryoprotectants

Fresh mature oocytes were immobilized on glass slides coated with poly-L-lysine and were mounted on the stage of an inverted microscope and equilibrated in phosphate-buffered saline (PBS). Dynamic oocyte volume changes resulted from the application of an osmotic challenge by replacing the initial PBS with a hyperosmotic PBS solution. For evaluation of water permeability, the hyperosmotic solution contained 200 mM sucrose (a non-permeant cryoprotectant). For evaluation of cryoprotectant permeability, a PBS solution containing 10 % DMSO and 10 % EG was used.

For water permeability, the relative cell volume change (shrinking) was fitted to a single exponential, and the estimated exponential rate constant k was used to calculate the osmotic permeability coefficient (P_f_) using the equation P_f_ = k (V_o_/A)(1/V_w_ (osm_out∞_) [14], where V_w_ is the molar volume of water, V_o_/A is the initial volume to the area ratio and osm_out∞_ is the final medium osmolarity after the applied osmotic gradient. For the cryoprotectant permeability (P_s_), the relative volume change after oocytes have reached their minimum value (reswelling) was fitted to a single exponential, and the estimated exponential rate constant k was used to calculate the cryoprotectant permeability using the equation P_s_ = k (V_o_/A) [14].

### Oocyte volume measurements

Oocyte volumes (V) were measured from 2D images obtained during the permeability assays (initial volume prior to the osmotic challenge V_o_ and final equilibrium volume after the osmotic challenge V_∞_). On a coverslip with 2 to 5 adhered oocytes, several pictures were taken at selected time points. For each experimental condition, four to five coverslips from different oocyte collections were measured (average of 20 oocytes analyzed per condition). Oocytes were assumed to have a spherical shape for the volume calculations.

### Experimental design

This study was approved by the Animal Care Committee of the National Veterinary Authority, following the appropriate European Union guidelines. Three experiments were designed.

#### Experiment 1

This experiment was performed to study the effect of CLA (100 μM trans-10, cis-12 octadecadienoic acid, Matreya, Pleasant Gap, Pennsylvania, USA [9]) on oocytes developmental competence after their exposure to cryoprotectants (sucrose, EG and DMSO [12]) and vitrification/warming (5 sessions). Four experimental groups were designed: (1) Control: cumulus-oocyte complexes (COC, *n* = 320) were matured (22 h) without supplementation; (2) CLA: COC (*n* = 350) were matured with CLA; (3) Control vitrified: COC (*n* = 355) were matured without supplementation and vitrified/warmed; (4) CLA vitrified: COC (*n* = 335) were matured with CLA and vitrified/warmed. Viable oocytes from all groups were inseminated with frozen-thawed semen, and embryo production was evaluated (cleavage and day 7/8 embryo rates).

#### Experiment 2

The effects of CLA and of the osmotic stress induced by the cryoprotectants on oocyte developmental competence (5 sessions) were studied. In this experiment, the vitrification process was mimicked without plunging the oocytes in liquid nitrogen (LN2). Thus, mature oocytes supplemented (CLA exposed, *n* = 247) or not (Control exposed, *n* = 240) with 100 μM CLA, were exposed to cryoprotectants as in experiment 1 but without freezing. Two non-exposed groups (Control *n* = 260 and CLA *n* = 253) were also tested. In each group, the embryo production rates and quality were evaluated.

#### Experiment 3

To investigate the effect of CLA on the fresh mature oocyte membrane permeability to water using sucrose (non-permeant cryoprotectant) and to EG and DMSO (permeant cryoprotectants), two experimental groups (CLA, *n* = 34; Control, *n* = 35) were constituted as above (3 sessions). Oocyte volumes and membrane water and cryoprotectant permeabilities were assessed.

### Statistical Analysis

Data representing 5 sessions of post-warming oocyte viability and 5 sessions of embryo production rates post-warming (experiment 1) or post-exposure to cryoprotectants (experiment 2) were analyzed using a MIXED procedure (proc mixed) of the Statistical Analysis Systems Institute (SAS Inst., Inc., Cary, NC, USA). The mixed linear model included the treatment as the fixed effect and the sessions as the random effect. In addition, the means for each treatment were calculated as well as the differences between the means and the respective *t*-test. For the oocyte membrane permeability data analysis, a Student’s *T*-test (proc ttest) was used. Differences were considered significant when *p* < 0.05.

### Results

#### Oocyte maturation and embryo production

**Fig. 1:**
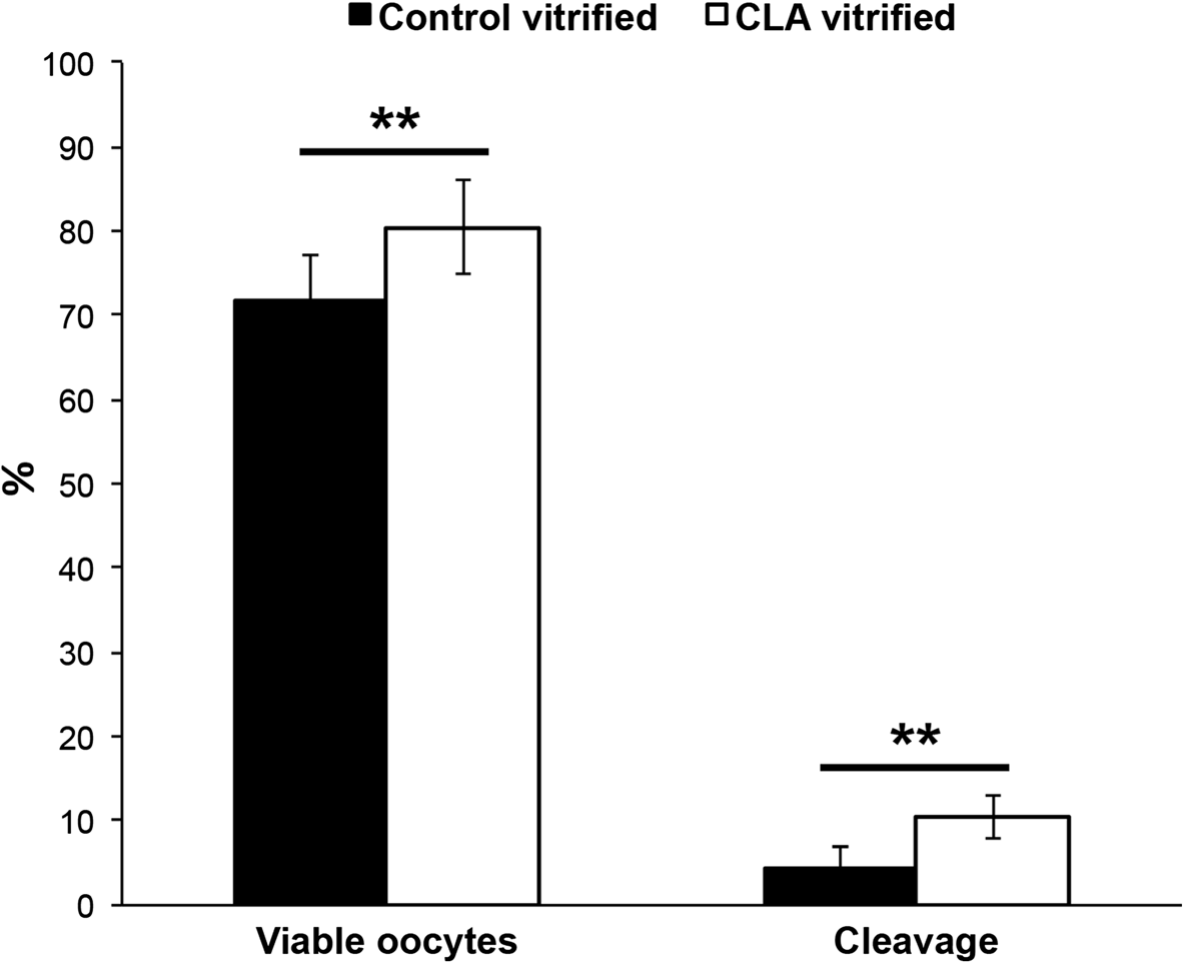
Effect of trans-10, cis-12 conjugated linoleic acid (CLA) on the cryosurvival of bovine oocytes. Viability was assessed by the integrity and morphology of thawed oocytes. Cleavage rate expressed as the percentage of cleaved embryos/viable oocytes. ** indicates statistical significance with a *p* < 0.01

In experiment 1, supplementation of oocyte culture medium with CLA improved the rate of viable COC that survived the vitrification process (CLA vitrified: 80.4 ± 5.6 % vs. Control vitrified: 71.7 ± 5.5 %, *p* = 0.01) and the cleavage rate (CLA vitrified: 10.4 ± 2.5 % vs. control vitrified: 4.5 ± 2.5 %, *p* = 0.01) (Fig. 1). As expected, the cleavage rate was lower (*p* < 0.0001) compared to fresh oocytes (CLA: 62.1 ± 3.0 % and Control: 62.2 ± 3.1 %). After vitrification/warming, no differences were identified between the CLA vitrified and control vitrified groups for the D7/8 blastocysts rate either considering viable oocytes or cleaved embryos (p > 0.05) for estimation (Table 1). This rate in vitrified oocytes was lower (*p* < 0.0001) compared to fresh groups when only taking into account the viable oocytes.

**Table 1:** Effect of trans-10, cis-12 conjugated linoleic acid (CLA) on the embryo production rates after vitrification/warming of bovine oocytes

Treatment	Viable oocytes (n)	D7/8 embryo/viable oocytes (%)	D7/8 embryo/cleaved embryos (%)
*Control*	235	21.2+/−2.0^a^	28.1+/−3.5
*CLA*	256	18.9+/−1.9^a^	27.4+/−3.9
*Control vitrified*	195	1.0+/−1.0^b^	14.4+/−15.8
*CLA vitrified*	207	3.0+/−1.0^b^	22.4+/−10.0

Data (mean ± SEM of 5 sessions, experiment 1) within columns with different superscripts are significantly different (*p* < 0.05)

In experiment 2, CLA mature oocytes exposed to cryoprotectants achieved a lower cleavage rate (*p* = 0.002) than those not exposed (Table 2). The Control exposed group presented the lowest D7/8 embryo rate (CLA group, *p* = 0.0004; Control group, *p* = 0.01; CLA exposed group, *p* = 0.07). Moreover, the Control exposed group had no grade 1 embryos (Table 3), showing lower rates than the Control (*p* = 0.01) and CLA (*p* = 0.001) groups. In contrast, no differences were identified between the CLA exposed group and the other groups. No differences were identified in the embryo grade 2 and 3 quality scores among groups.

**Table 2:** Effect of trans-10, cis-12 conjugated linoleic acid (CLA) on the embryo production rates of bovine oocytes exposed to osmotic stress by cryoprotectants without vitrification

Treatment	Inseminated oocytes (n)	Cleavage (%)	D7/8 embryo (%)
*Control*	209	70.5+/−5.1^ab^	25.3+/−4.5^a^
*CLA*	194	78.9+/−5.2^a^	30.8+/−4.7^a^
*Control exposed*	207	71.8+/−5.1^ab^	13.3+/−4.4^b^
*CLA exposed*	202	64.4+/−5.2^b^	21.5+/−4.7^ab^

Data (mean ± SEM of 5 sessions, experiment 2) within columns with different superscripts are significantly different (*p* < 0.05)

**Table 3:** Distribution of embryo morphological quality after oocyte culture with trans-10, cis-12 conjugated linoleic acid isomer (CLA) and exposure to osmotic stress by cryoprotectants without vitrification

Treatment	Number	Grade 1 (%)	Grade 2 (%)	Grade 3 (%)
*Control*	37	29.7+/−6.9^a^	10.8+/−5.4	57.1+/−8.3
*CLA*	38	39.5+/−6.9^a^	5.2+/−5.4	53.3+/−8.2
*Control exposed*	20	0+/−9.4^b^	20.0+/−7.4	79.2+/−11.2
*CLA exposed*	26	19.2+/−8.3^ab^	19.3+/−6.5	59.7+/−10.2

Data (mean ± SEM of 5 sessions, experiment 2) within columns with different superscripts are significantly different (*p* < 0.05)

### Oocyte dimensions and membrane permeability

In experiment 3, the radius of Control bovine oocytes in an isotonic solution was 53.3 ± 3.7 μm (*n* = 34; mean ± SEM). The radius of oocytes matured in CLA in an isotonic solution was not different from control oocytes and was 54.8 ± 0.63 μm (*n* = 35; mean ± SEM).

**Fig. 2:**
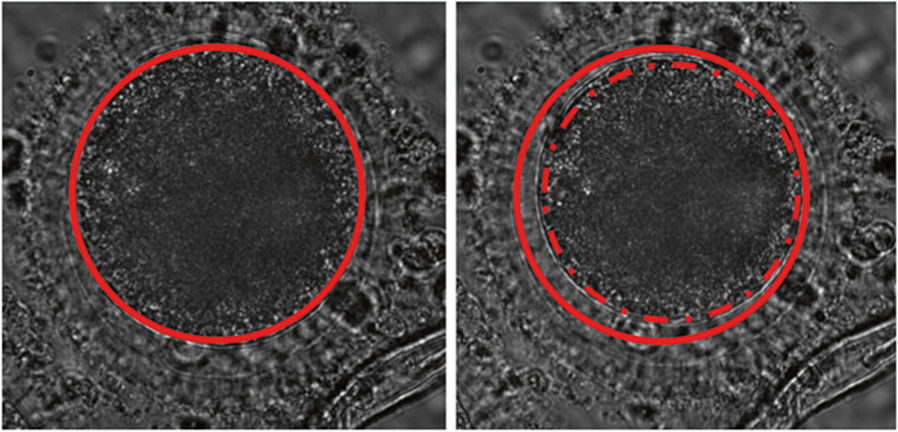
Oocyte volumes during permeability assays. Representative illustration of oocyte with initial equilibrium volume Vo (left panel) and final equilibrium volume V∞ (right panel) after an osmotic challenge with sucrose to evaluate the membrane permeability to water

**Fig. 3:**
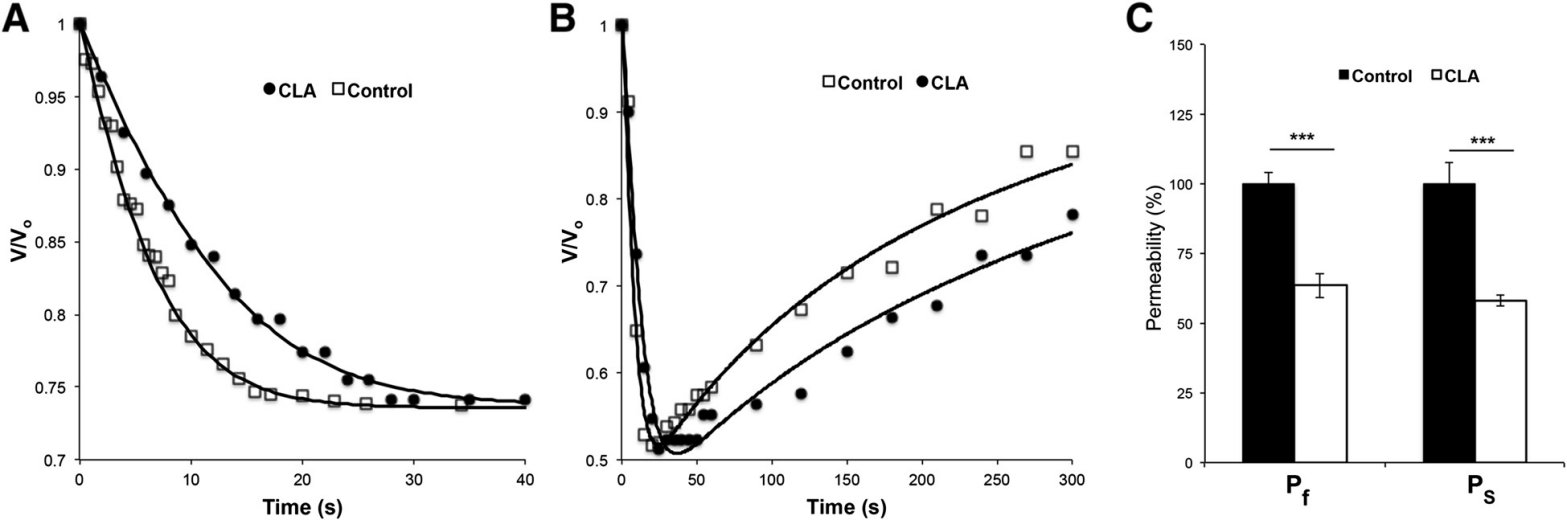
Effect of trans-10, cis-12 conjugated linoleic acid (CLA) on oocyte membrane permeability to water and cryoprotectants. **a** Time course of the oocyte volume change after the addition of sucrose (200 mM) to the extracellular media (oocyte shrinkage). The rate at which the oocyte shrinks is visibly slower when oocytes are matured in the presence of CLA. **b** Time course of the oocyte volume change after the addition of 10 % EG and 10 % DMSO to the extracellular media. The first water outflow (shrinkage) is followed by an influx of cryoprotectant with consequent oocyte reswelling. The rate of cryoprotectant influx is slower for oocytes matured in the presence of CLA. **c** Oocyte membrane permeability (%, relative to control) for oocytes control and oocytes matured with CLA. P_f_, permeability to water. P_s_, permeability to cryoprotectants (10 % EG and 10 % DMSO). *** indicates significant difference (*p* < 0.001)

For evaluation of the osmotic water permeability coefficient P_f_, Control and CLA mature oocytes were subjected to a hyperosmotic gradient of 200 mM of the non-permeant cryoprotectant sucrose. Typical volume changes can be observed from the pictures of oocytes before (initial volume V_o_) and after the hyperosmotic shock when oocytes have reached their new final equilibrium volume V_∞_ (Fig. 2). As expected, in both groups of oocytes, a decrease in volume due to water outflow could be detected. However, observing the respective traces of relative volume change (V/V_o_) (Fig. 3a), the rate at which the oocyte shrinks is visibly different (*p* < 0.001) between the Control and CLA oocytes, where the estimated P_f_ (x10^−3^ cm s^−1^, mean ± SEM) values are 14.35 ± 0.55 (*n* = 10) for the Control and 9.13 ± 0.62 (*n* = 10) for the CLA group.

For evaluation of the cryoprotectant permeability, Control and CLA mature oocytes were subjected to a hyperosmotic gradient by the addition of a solution containing 10 % DMSO and 10 % EG. Because these cryoprotectants are permeable, the water flux (due to the osmotic gradient) and cryoprotectant fluxes (due to their concentration gradients) are expected to occur simultaneously and to induce cell volume changes. Typical relative volume changes can be observed in Fig. 3b, where a first decrease in cell volume due to water outflow is observed during the first 20–40 s, followed by a subsequent increase due to the influx of cryoprotectant. These volume changes are slower for CLA oocytes. The estimated P_S_ (x10^−6^ cms^−1^, mean ± SEM) values are 11.39 ± 0.84 (*n* = 20) for the Control and 6.62 ± 0.23 (*n* = 20) for the CLA groups (Fig. 3b). Similar permeability values were reported for control oocytes [15], despite using a different experimental approach.

Thus, a significant slowing of the water and cryoprotectant flux rates was observed (*p* < 0.001), corresponding to a 37 % decrease in water permeability and a 42 % decrease in cryoprotectant permeability due to CLA supplementation (Fig. 3c).

## Discussion

Cryopreservation technology of the female gamete remains imperfect [1, 2], although the birth of live babies [16] and calves [17] from cryopreserved oocytes has been achieved. However, to date, only approximately 500 babies have been born from cryopreserved oocytes, as opposed to tens of thousands that have developed from frozen embryos [16]. Several critical points during oocyte cryopreservation have been identified, specifically the opposite effects of the cooling rate and the sample volume achieved. Increasing the cooling rate improves the survival rate, and the smaller the sample volume the higher the probability of a successful vitrification. Furthermore, during cryopreservation, the survival rate can be modified by varying the viscosity of the medium and/or the cryoprotectants and other additives used [1, 2, 18]. The results presented in this study reflect the dimensions of this multifaceted problem. The low blastocyst rates achieved here after IVF of vitrified/warmed bovine oocytes are near the range found by other authors [19–21]. However, recent reports claim higher and more promising values, up to 54.4 % [22], thus opening new opportunities for successful application of oocyte cryopreservation techniques. The use of heifer oocytes and of OPS devices in the present work contributed to the low embryo rates. According to recent data, improved cryosurvival is obtained by using the cryotop technique and oocytes from older cows [17, 18, 20].

The osmotic stress induced by cryopreservation has been implicated in the alteration of the zona pellucida and of the ooplasm [18, 23]**,** causing a series of ultrastructural and functional damage that might compromise oocyte developmental competence [24]. In this work, CLA media supplementation improved the viability of vitrified/warmed COC, helping to lessen this damage. Moreover, the positive effect of CLA in oocyte developmental competence was also recorded after the osmotic stress induced by cryoprotectants without vitrification. These oocytes (CLA exposed group) had similar D7/D8 embryo rates and quality to those not exposed to cryoprotectant toxicity. Conversely, oocytes from the Control exposed group presented the lowest D7/8 embryo rates and produced no good quality embryos (Table 2 and 3). These observations further support the assessment that this CLA isomer improves the resistance of mature oocytes to the osmotic stress during the cryopreservation process. As previously reported [11, 25], during COC culture in CLA supplemented medium, this isomer was accumulated by oocytes and by cumulus cells, changing their fatty acid content and profiles and prostaglandins synthesis during the maturation process. Although presenting similar oocyte nuclear maturation rates, CLA oocytes had smaller fat areas. The lipid content reduction and the fatty acid profile modifications induced by CLA either in the oocyte or in the cumulus cells during COC maturation [11, 25, 26] contribute to the increased oocyte resistance to cryopreservation. Others have reported that this isomer reduced the lipid content of in vitro produced bovine embryos through the reduction of the gene expression of 1-acylglycerol 3-phosphate 0-acyltransferase enzyme [27]. Improved cryosurvival of CLA supplemented embryos both in cattle and in sheep was also documented [9, 10, 28].

Furthermore, the plasma membrane permeability is another important factor for the tolerance of cells to cryopreservation because it may modulate several major forms of cell injury caused by the cryopreservation process, such as cryoprotectant toxicity and drastic volume changes due to the induced osmotic stress [23]. Recent results demonstrated that CLA interferes in the permeability and fluidity of adipose cells plasma membranes [6]. The potential effect of another polyunsaturated fatty acid, linoleic acid (LA), in membrane fluidity and cryosurvival of in vitro produced bovine embryos was also speculated [29]. Moreover, in obese Zucker rats fed CLA, consistently low adipose membrane permeability indicated that in these membranes, permeation occurred mostly via the lipid bilayer [6]. The observed decrease in membrane permeability was correlated with the simultaneous decrease in membrane fluidity caused by CLA incorporation into membrane phospholipids. As presented for the first time in this study, CLA supplementation during bovine oocytes maturation also lowers their membrane permeability. Our results show a CLA effect (*p* < 0.001) on the water outflow rate induced by an osmotic challenge with sucrose, a non-permeant cryoprotectant currently used in oocyte cryopreservation media [17, 21]. Indeed, for CLA treated oocytes, the slower shrinkage and lower water permeability suggest that bovine oocytes matured in the presence of CLA are more resistant to osmotic stress, which would help to minimize their damage during cryopreservation. Furthermore, a slower influx of the 10 % EG and 10 % DMSO cryoprotectant solutions is also observed for bovine oocytes matured in the presence of CLA. These results may be due to CLA incorporation in the oocyte membrane, thus affecting membrane fluidity. Therefore, the use of CLA should be considered for the improvement of oocyte cryopreservation.

When crossing the plasma membrane, water and cryoprotectants can follow two different pathways, depending on the developmental stage of the cell and on the cryoprotectant used: diffusion through the lipid bilayer and/or facilitated diffusion through channels [15]. On oocyte membranes, water and cryoprotectants move mainly through the lipid bilayer and, as expected, the flow rates are highly dependent on temperature. Shrinkage and reswelling of oocytes are much slower at 15 °C compared to 25 °C due to the lower conductivity of water and cryoprotectants in the lower temperature range [15, 18]. Improved results can be obtained by loading cryoprotectant at a hypothermic temperature, typically at room temperature [30]. At this lower temperature, longer periods of exposure to the cryopreservation solution are necessary to dehydrate the cell and allow the cryoprotectant to penetrate. We hypothesize that CLA incorporation into the oocyte membrane and the resulting delay of water and cryoprotectant fluxes, mimics the process conducted at lower temperatures known to improve cryopreservation.

On the other hand, unlike mouse oocytes, in bovine oocytes, a smaller proportion of water moves via channels [15]. In this case, CLA membrane incorporation alters the channel/lipid environment, influencing channel activity. Overall, reducing channel and bilayer permeation modulates the permeation rate of water and cryoprotectants with a positive outcome on oocyte survival.

The extent of the cryoinjuries is highly variable, depending on the oocyte maturation stage and species [1, 3, 4, 19]. Higher membrane permeability improves cell dehydration, namely, the influx of cryoprotectants before cooling and their removal after warming. Reports showing that bovine oocytes are less tolerant to cryopreservation than mouse oocytes, although presenting higher membrane water and cryoprotectant permeability, [27] contradict this theory. Accordingly, our results indicate that it is the reduction of cryoprotectants and the water flux rates (osmotic shrinking and re-swelling) that favors cell survival after cryopreservation.

## Conclusions

Our results show that CLA supplementation in the maturation medium modulates oocyte membrane water and cryoprotectant permeability. By slowing the fluxes of water and of cryoprotectants, CLA contributes to improved oocyte cryosurvival and post-thawed viability. These results provide a tool for minimizing damage during the addition and removal of cryoprotectants, maintaining oocyte normal physiology throughout the vitrification and warming processes.

## Abbreviations

BSA: Bovine serum albumin
CLA: Trans-10, cis-12 conjugated linoleic acid
COC: Cumulus oocyte complexes
DMSO: Dimethyl sulfoxide
EG: Ethylene glycol
FCS: Fetal calf serum
FCT: Portuguese Foundation for Science and Technology
GSH: Glutathione
HM: Holding medium
IVF: in vitro fertilization
LN2: Liquid nitrogen
osm_out∞_: Final medium osmolarity after the applied osmotic gradient
OPS: Open pulled straw
PBS: Phosphate-buffered saline
P_f_: Osmotic water permeability coefficient
Ps: Cryoprotectant permeability
SOCS: Superovulated estrus cow serum
SOF: Synthetic oviductal fluid
TCM 199: Tissue culture medium 199
V: Oocyte volume
V_o_/A: Initial volume to area ratio
V_w_: Molar volume of water
